# Emergence of eravacycline heteroresistance in carbapenem-resistant *Acinetobacter baumannii* isolates in China

**DOI:** 10.3389/fcimb.2024.1356353

**Published:** 2024-03-27

**Authors:** Yi-tan Li, Xian-di Chen, Ying-yi Guo, Shan-wen Lin, Ming-zhen Wang, Jian-bo Xu, Xiao-hu Wang, Guo-hua He, Xi-xi Tan, Chao Zhuo, Zhi-wei Lin

**Affiliations:** ^1^ Key Laboratory of Respiratory Disease, People’s Hospital of Yangjiang, Yangjiang, China; ^2^ Guangzhou Institute of Respiratory Health, First Affiliated Hospital of Guangzhou Medical University, Guangzhou, China

**Keywords:** eravacycline, carbapenem-resistant *Acinetobacter baumannii*, heteroresistance, AdeABC efflux pump, IS*Aba1*

## Abstract

Carbapenem-resistant *Acinetobacter baumannii* (CRAB) is resistant to almost all antibiotics. Eravacycline, a newer treatment option, has the potential to treat CRAB infections, however, the mechanism by which CRAB isolates develop resistance to eravacycline has yet to be clarified. This study sought to investigate the features and mechanisms of eravacycline heteroresistance among CRAB clinical isolates. A total of 287 isolates were collected in China from 2020 to 2022. The minimum inhibitory concentration (MIC) of eravacycline and other clinically available agents against *A. baumannii* were determined using broth microdilution. The frequency of eravacycline heteroresistance was determined by population analysis profiling (PAP). Mutations and expression levels of resistance genes in heteroresistant isolates were determined by polymerase chain reaction (PCR) and quantitative real-time PCR (qRT-PCR), respectively. Antisense RNA silencing was used to validate the function of eravacycline heteroresistant candidate genes. Twenty-five eravacycline heteroresistant isolates (17.36%) were detected among 144 CRAB isolates with eravacycline MIC values ≤4 mg/L while no eravacycline heteroresistant strains were detected in carbapenem-susceptible *A. baumannii* (CSAB) isolates. All eravacycline heteroresistant strains contained OXA-23 carbapenemase and the predominant multilocus sequence typing (MLST) was ST208 (72%). Cross-resistance was observed between eravacycline, tigecycline, and levofloxacin in the resistant subpopulations. The addition of efflux pump inhibitors significantly reduced the eravacycline MIC in resistant subpopulations and weakened the formation of eravacycline heteroresistance in CRAB isolates. The expression levels of *adeABC* and *adeRS* were significantly higher in resistant subpopulations than in eravacycline heteroresistant parental strains (*P* < 0.05). An IS*Aba1* insertion in the *adeS* gene was identified in 40% (10/25) of the resistant subpopulations. Decreasing the expression of *adeABC* or *adeRS* by antisense RNA silencing significantly inhibited eravacycline heteroresistance. In conclusion, this study identified the emergence of eravacycline heteroresistance in CRAB isolates in China, which is associated with high expression of AdeABC and AdeRS.

## 1 Introduction

*Acinetobacter baumannii* is a major pathogen responsible for nosocomial infections, primarily causing ventilator-associated pneumonia, bloodstream infections, and skin and soft tissue infections (Durante-Mangoni and Zarrilli, 2011). In recent years, due to the widespread use of carbapenem antibiotics, an increasing number of carbapenem-resistant *Acinetobacter baumannii* (CRAB) strains have been reported. CRAB strains typically exhibit multidrug resistance, leaving a few options, including ampicillin-sulbactam, polymyxins B, and tigecycline, to treat CRAB infections (Pogue et al., 2013). In recent years, polymyxins B and tigecycline-resistant isolates have been increasingly reported in CRAB isolates. Thus, there is an urgent need for novel antibiotics to treat CRAB infections in clinical practice.

Eravacycline is a novel synthetic fluorocycline antimicrobial agent that was modified from tigecycline. It is active against antibiotic-resistant pathogens, such as multidrug-resistant *A. baumannii* strains, methicillin-resistant *Staphylococcus aureus*, vancomycin-resistant *Enterococci*, and carbapenem-resistant *Enterobacteriaceae* (Monogue et al., 2016; Li et al., 2022). Notably, eravacycline is four times more effective than tigecycline against multidrug-resistant *A. baumannii* (Zhanel et al., 2016). Eravacycline was approved for the treatment of complicated intra-abdominal infections in adults based on the success of two phase III clinical trials, IGNITE 1 and IGNITE 4, by the U.S. Food and Drug Administration (FDA) in 2018 (Wang et al., 2021a). Eravacycline is an intravenous and oral medication with the potential to treat CRAB infections. In an analysis of phase III clinical trials, favorable clinical and microbiological responses were observed for eravacycline against CRAB. In patients with complicated intra-abdominal infections caused by multidrug-resistant *A. baumannii*, of which main strains were CRAB, eravacycline-treated patients achieved a 100% clinical and microbiologic cure (Poulakou et al., 2018). The common adverse events of eravacycline reported in phase I-III studies included infusion-site reactions and gastrointestinal effects such as nausea, vomiting and diarrhea, and it may cause the same adverse events as other tetracyclines (Yusuf et al., 2021).

Heteroresistance is a form of antibiotic resistance in which a bacterial isolate harbors a minority-resistant subpopulation that coexists with a majority-susceptible population. It is difficult to detect using standard antimicrobial susceptibility testing and often results in antibiotic treatment failure (Choby et al., 2021). While the clinical relevance of antibiotic heteroresistance is unclear, there is increasing evidence that heteroresistance causes treatment failure due to the selection of resistant subpopulations after antibiotic treatment (Wen et al., 2020). Thus, the detection and characterization of heteroresistance can provide appropriate therapeutic guidance for antibiotic treatment. Eravacycline heteroresistance is found in only a few bacteria, including *Staphylococcus aureus*, *Enterococcus faecalis*, and *Klebsiella pneumoniae* (Zheng et al., 2018b; Wang et al., 2020; Wen et al., 2020), but has not yet been investigated in CRAB.

This study sought to investigate the phenomenon and mechanism of eravacycline heteroresistance in CRAB clinical isolates from China. The frequency and characteristics of eravacycline heteroresistant strains were analyzed. Molecular sequencing, quantitative real-time PCR (qRT-PCR), and *in vitro* functional tests were performed to explore the incidence and underlying mechanism of eravacycline heteroresistance in CRAB isolates. These findings will inform the current understanding of eravacycline resistance mechanism.

## 2 Materials and methods

### 2.1 Bacterial strains, growth conditions, and antibiotics

From January 2020 to December 2022, 287 non-duplicate *A. baumannii* isolates, including 147 CRAB and 140 carbapenem-susceptible *A. baumannii* (CSAB) isolates, were collected from various clinically sampled infections of inpatients at Yangjiang People’s Hospital, a tertiary hospital with 2000 beds in Guangdong Province of China. Bacterial species were identified using a VITEK 2 compact system (BioMérieux, Marcy l’Etoile, France). All strains in this study were cultured on Mueller-Hinton (MH) broth (Oxoid, Basingstoke, UK), or in MH agar plates and Luria-Bertani (LB) broth (Oxoid, Basingstoke, UK) at 37°C. All procedures performed were approved by the Ethical Committee of Yangjiang People’s Hospital and were in accordance with the 1964 Helsinki Declaration and its later amendments. The novel fluorocycline antibiotic, eravacycline (catalog no. HY-16980A), was purchased from MedChem Express (MCE, Shanghai, China). The other antimicrobials were purchased from Meilunbio (Dalian, China). *Escherichia coli* ATCC 25922 was used for quality control.

### 2.2 Antimicrobial susceptibility testing

The minimum inhibitory concentrations (MICs) of eravacycline and several common antibiotics were determined using the broth microdilution method recommended by Clinical and Laboratory Standards Institute (CLSI) guidelines (CLSI-M100-S30). As *A. baumannii* MIC breakpoints for eravacycline and tigecycline have not yet been established by CLSI and FDA, this study categorized the MIC values into three levels described by prior studies (Marchaim et al., 2014; Abdallah et al., 2015): ≤2 mg/L (susceptible), 4 mg/L (intermediate), and ≥8 mg/L (resistant). The susceptibility results for other antibiotics, including meropenem, levofloxacin, gentamicin, cefepime, polymyxin B, tetracycline, doxycycline, and minocycline, were determined based on CLSI breakpoints for each antibiotic. In this study, CRAB strains were defined as those with an imipenem and/or meropenem MIC ≥16 mg/L, while CSAB strains with imipenem and meropenem MICs were both ≤4 mg/L (Fu et al., 2010).

### 2.3 Population analysis profiles

Population analysis profiling (PAP) was performed as described previously (Abe et al., 2022). The method was used as a reference to investigate eravacycline heteroresistance among 283 clinical *A. baumannii* isolates with eravacycline MIC values ≤4 mg/L. In brief, 50 μL aliquots of cell suspension (corresponding to a 0.5 McFarland standard for *A. baumannii* cultures grown on blood agar plates for 24 h at 37°C, 1.0–1.5 × 10^8^ CFU/mL) were spread onto MH agar plates with or without eravacycline (1, 2, 4, 8, 16, and 32 mg/L). The plates were incubated for 24 h at 37°C and the number of colonies was counted. Based on the breakpoint of eravacycline MIC values for *A. baumannii*, eravacycline heteroresistance was defined as an eravacycline-susceptible isolate (MIC ≤ 4 mg/L) with subpopulations growing in the presence of ≥8 mg/L eravacycline at a detection threshold of 20 CFU/mL. Three colonies were selected from the 8 mg/L eravacycline concentration of the PAP test and categorized as the resistant subpopulations of each eravacycline-heteroresistant isolate (name as: strain-RS). The eravacycline-heteroresistant parental strain was considered as the susceptible subpopulation (name as: strain-HP) given that the majority subpopulations are susceptible to eravacycline (Band and Weiss, 2019). The resistant subpopulations of eravacycline MICs were reassessed after serial passaging on antibiotic-free medium to evaluate the stability of the heteroresistant phenotype.

### 2.4 Polymerase chain reaction for multilocus sequence typing, carbapenemase, and genetic mutations in the resistance genes

Total DNA from eravacycline heteroresistant parental strains and their resistant subpopulations were extracted using lysis buffer for microorganisms. Polymerase chain reaction (PCR) was performed using PCR Mastermix (Thermo Fisher Scientific, Waltham, MA) according to the manufacturer’s instructions. Multilocus sequence typing (MLST) of the strains was determined by PCR and sequence alignment, as described previously (Huang et al., 2016). The carbapenemase resistance genes, *bla*_OXA_, *bla*_KPC_, *bla*_IMP_, *bla*_VIM_, *bla*_SIM,_ and *bla*_NDM-1_ were detected by PCR (Hassan et al., 2021). Genetic mutation of the RND efflux pump regulators, *adeR*, *adeS*, *adeL*, and *adeN*, and the ribosomal protein S10 encoding gene, *rpsJ*, were detected by PCR amplification and sequence alignment. The primers used for PCR are listed in **Supplementary Tables 1–3.**

### 2.5 Efflux pump inhibitors assays

The role of the efflux pump in the eravacycline heteroresistance of CRAB isolates was detected using the efflux pump inhibitors (EPIs), Phe-Arg-β-naphthylamide (PaβN; MCE, Shanghai, China), and carbonyl cyanide m-chlorophenylhydrazone (CCCP; MCE, Shanghai, China). Eravacycline MIC was determined using the agar dilution method in the presence and absence of PAβN (50 mg/L) or CCCP (16 mg/L) in resistant subpopulations of the heteroresistant isolates, as previously described (Jo and Ko, 2021). Efflux inhibition was defined as a ≥4-fold decrease in the MICs from their original values in the presence of EPI (Tambat et al., 2022). PAP assay with PAβN (50 mg/L) or CCCP (16 mg/L) added to the MH agar plates was used to validate the potential function of the efflux pump in eravacycline heteroresistance.

### 2.6 Quantitative real-time PCR analysis

The transcriptional levels of the RND-type efflux pump genes (*adeB, adeG*, and *adeJ*) and their regulators (*adeS, adeL*, and *adeN*) were quantified in eravacycline heteroresistant parental strains and resistant subpopulations using qRT-PCR as described previously (Yoon et al., 2013), with the primers listed in **Supplementary Table 4.** Since the three genes, *adeA*, *adeB*, and *adeC*, of the efflux pump, *adeABC*, are located within the same operon, *adeB* was considered representative of *adeABC* efflux pump expression in this study (Ju et al., 2021). The other genes shared the same principle. In brief, overnight cultures of the bacterial strains were diluted 1:100 in 10 mL of LB broth and incubated at 37°C with 220 rpm shaking until the growth reached a logarithmic phase. Total RNA was extracted using a Bacteria RNA Extraction Kit (Vazyme, Nanjing, China), and cDNA was synthesized with a HiScriptIII-RT SuperMix for qRT-PCR kit (Vazyme, Nanjing, China) according to the manufacturer’s instructions. The qRT-PCR was performed using a ChamQ Universal SYBR qRT-PCR Master Mix (Vazyme, Nanjing, China) in a LightCycler480II system (Roche, Basel, Switzerland), with the following parameters: 1 cycle at 95°C for 30 s, followed by 45 cycles of 95°C for 10 s and 60°C for 30 s. The internal control gene, *rpoB*, was used to normalize the expression of each candidate gene. Each sample was run in triplicate. Threshold cycle (Ct) numbers were determined using the qRT-PCR system software, and the relative transcription levels of all strains were calculated using the 2^−ΔΔCt^ method. CRAB-9-HP was used as the reference strain (expression = 1.0). The expression levels of the resistant subpopulation target genes were compared with their parental strains.

### 2.7 AdeABC and AdeRS associated with heteroresistance were verified by antisense RNA silencing

To confirm the role of AdeABC and AdeRS in the eravacycline heteroresistance of CRAB isolates, the efflux pump genes were silenced by antisense RNA (asRNA) in strains with a high expression of *adeABC* or *adeRS* in resistant subpopulations and their parental strains. For silencing *adeABC*, an *adeABC* asRNA expression plasmid was constructed by first amplifying the Shine–Dalgarno sequence plus ∼148 nt downstream of the start codon of *adeABC* and then inserting the segment between the HindIII and BamHI sites downstream of the isopropyl β-D-thiogalactoside (IPTG; MCE, Shanghai, China)-inducible promoter in pHN679 (Zheng et al., 2018a). For generating expression plasmids of *adeRS* asRNA, the Shine–Dalgarno sequence plus ∼146 nt downstream of the start codon of *adeRS* was amplified. The asRNA expression plasmids, pAS*adeABC* and pAS*adeRS*, were verified by PCR and DNA sequencing, separately transformed into resistant subpopulations and their parental strains by electroporation, and then verified again using PCR. The pHN679 was also introduced into the isolates as a vector control. Strains used for asRNA silencing are listed in **Supplementary Table 5** and primers used for the construction and validation of transformed strains are listed in **Supplementary Table 6**. The silencing efficacy of the target genes was measured using qRT-PCR as described above. For RNA isolation of the *adeABC*- and *adeRS*-silenced strains, the bacteria were pre-treated with 1 mM IPTG to induce expression of the asRNA silencing plasmids. The eravacycline MIC for the *adeABC*- and *adeRS*-silenced resistance subpopulations was performed using the broth microdilution method. The frequency of eravacycline heteroresistance in the *adeABC*- and *adeRS*-silenced parental strains was determined using the PAP test.

### 2.8 Statistical analysis

We used Student’s *t*-test for continuous variables, chi-square analysis for categorical variables. All tests were performed in IBM SPSS Statistics (version 22.0; IBM, Chicago, USA). *P*-values <0.05 are regarded as statistically significant.

## 3 Results

### 3.1 Frequency of eravacycline heteroresistance in clinical CRAB and CSAB isolates

The susceptibilities of 147 CRAB and 140 CSAB strains to eravacycline and other antibiotics are summarized in **Supplementary Table 7**. Eravacycline, tigecycline and polymyxins B exhibited excellent antimicrobial activity against CRAB. The MIC_90_ for eravacycline (1 mg/L) was lower than tigecycline (2 mg/L) and polymyxins B (2 mg/L) against these strains. Eravacycline heteroresistance was determined using the PAP test in 144 CRAB and 139 CSAB strains with an eravacycline MIC ≤4 mg/L. While 25 CRAB strains (17.36%) exhibited heteroresistance to eravacycline, no heteroresistant strains were detected in CSAB isolates. Furthermore, the frequency of eravacycline heteroresistance in CRAB isolates was increased with the patent of MIC value (**Table 1**).

**Table 1:** The frequency of eravacycline heteroresistance in clinical CRAB and CSAB isolates.

MIC of CRAB isolates (mg/L)	PAP test^a^		Heteroresistant rate (%)	MIC of CSAB isolates (mg/L)	PAP test		Heteroresistant rate (%)
	Positive	Negative			Positive	Negative	
≤0.06 (4^b^)	0	4	0	≤0.06 (103)	0	103	0
0.12–0.25 (124)	18	106	14.52	0.12–0.25 (28)	0	28	0
0.5–1 (12)	5	7	41.67	0.5–1 (5)	0	5	0
2–4 (4)	2	2	50.00	2–4 (3)	0	3	0
Total (144)	25	119	17.36	Total (139)	0	139	0

a Eravacycline heteroresistance is defined as an eravacycline-susceptible isolate (MIC ≤ 4 mg/L) with subpopulations growing in the presence ≥8 mg/L eravacycline, with a detection threshold of 20 CFU/mL.

b Number of strains with different MIC values.

Colonies selected from the 8 mg/L eravacycline PAP test concentration were categorized as the resistant subpopulations of each eravacycline-heteroresistant isolate. The parental strains of eravacycline-heteroresistant isolates were considered susceptible subpopulations in this study since the majority subpopulations were susceptible to eravacycline. The resistant subpopulations had 8- to 128-fold higher eravacycline MICs than the parental strains (**Table 2**). The eravacycline MICs of resistant subpopulations were 4- to 8-fold lower after 15 passages on antibiotic-free medium and were reversed to susceptible level after 30 passages (**Supplementary Table 8**). These findings suggest that these subpopulations lacked stable mutations conferring eravacycline resistance and could be reversed to the susceptible phenotype after removal of the antibiotic pressure.

**Table 2:** Characteristics of eravacycline heteroresistance in CRAB clinical isolates.

Strains	Gender	Age	Source	Department	Outcomes	Eravacycline MIC (mg/L)		MLST^b^	Carbapenemase gene	
						HP strains^a^	RS strains			
CRAB-5	male	82	Wound secretion	Department of Trauma and Orthopaedics	Cure	0.5	32	1959	*bla*_OXA-23_, *bla*_OXA-24_	
CRAB-9	male	64	Wound secretion	Department of neurosurgery	Cure	0.25	16	208	*bla*_OXA-23_	
CRAB-11	male	72	Sputum	Intensive care unit	Cure	1	64	92	*bla*_OXA-23_, *bla*_OXA-24_	
CRAB-14	male	82	Wound secretion	Intensive care unit	Cure	0.25	32	208	*bla*_OXA-23_, *bla*_OXA-24_	
CRAB-21	male	67	Sputum	Department of neurosurgery	Cure	0.12	16	208	*bla*_OXA-23_, *bla*_OXA-48_	
CRAB-24	male	72	Sputum	Intensive care unit	Cure	1	16	195	*bla*_OXA-23_, *bla*_OXA-24_	
CRAB-38	male	65	Wound secretion	Department of burn plastic surgery	Death	4	64	208	*bla*_OXA-23_, *bla*_OXA-24_	
CRAB-44	male	71	Sputum	Intensive care unit	Abandoning Treatment	0.25	32	208	*bla*_OXA-23_, *bla*_OXA-48_	
CRAB-49	female	58	Urine	Department of urinary surgery	Cure	0.5	16	208	*bla*_OXA-23_	
CRAB-50	male	67	Sputum	Intensive care unit	Cure	0.25	32	195	*bla*_OXA-23_, *bla*_OXA-24_	
CRAB-53	female	74	Wound secretion	Department of burn plastic surgery	Cure	0.25	16	208	*bla*_OXA-23_, *bla*_OXA-24_	
CRAB-58	male	45	Blood	Intensive care unit	Death	0.12	16	208	*bla*_OXA-23_, *bla*_KPC_	
CRAB-64	female	67	Sputum	Intensive care unit	Cure	0.25	32	208	*bla*_OXA-23_, *bla*_OXA-24_	
CRAB-68	male	68	Sputum	Intensive care unit	Death	0.25	16	208	*bla*_OXA-23_	
CRAB-77	male	74	Sputum	Intensive care unit	Cure	0.12	16	451	*bla*_OXA-23_, *bla*_OXA-48_	
CRAB-82	female	83	Urine	Department of Neurology	Cure	0.25	32	208	*bla*_OXA-23_, *bla*_OXA-24_	
CRAB-84	male	68	Sputum	Department of neurosurgery	Cure	0.25	16	208	*bla*_OXA-23_, *bla*_OXA-24_	
CRAB-87	male	68	Wound secretion	Department of Trauma and Orthopaedics	Cure	0.12	16	208	*bla*_OXA-23_	
CRAB-90	male	71	Sputum	Department of neurosurgery	Cure	0.25	16	208	*bla*_OXA-23_, *bla*_OXA-58_	
CRAB-92	female	64	Sputum	Intensive care unit	Cure	0.25	16	208	*bla*_OXA-23_	
CRAB-102	male	68	Sputum	Department of neurosurgery	Death	0.25	32	208	*bla*_OXA-23_, *bla*_OXA-24_	
CRAB-108	female	53	Wound secretion	Department of burn plastic surgery	Cure	0.25	32	195	*bla*_OXA-23_, *bla*_OXA-24_	
CRAB-113	male	68	Wound secretion	Department of neurosurgery	Cure	0.25	32	208	*bla*_OXA-23_, *bla*_OXA-24_	
CRAB-127	male	66	Wound secretion	Department of burn plastic surgery	Cure	2	32	208	*bla*_OXA-23_, *bla*_OXA-58_	
CRAB-139	male	73	Sputum	Intensive care unit	Cure	1	16	191	*bla*_OXA-23_, *bla*_IMP_	

a HP strains, heteroresistant parental strains; RS strains, resistant subpopulations of heteroresistance strains.

b MLST, multilocus sequence typing of Oxford scheme.

### 3.2 Characteristics of eravacycline heteroresistance in CRAB isolates

The epidemiological and molecular characteristics of eravacycline heteroresistant strains are listed in **Table 2**. These strains were primarily shown to infect individuals >60 years of age (n = 22, 88%). The heteroresistant strains came from various specimens, including sputum (n = 13, 52%), wound secretions (n = 9, 36%), urine (n = 2, 8%), and blood (n = 1, 4%) that were primarily obtained from the intensive care unit (ICU) (n = 11, 44%), department of neurosurgery (n = 6, 24%) and department of burn plastic surgery (n = 4, 16%). Four patients infected with the heteroresistant strains had a fatal clinical outcome (n = 4, 16%). MLST revealed six sequence types (ST) in 25 eravacycline heteroresistant strains, with ST208 (n = 18, 72%) being the most prevalent. The predominant carbapenemase genes among these strains were *bla*_OXA-23_ (n = 25, 100%) and *bla*_OXA-24_ (n = 13, 52%) (**Table 2**).

### 3.3 Change in the antimicrobial activity to common antibiotics against resistant subpopulations

The *in vitro* antimicrobial activity of common antibiotics against resistant subpopulations and parental strains were compared to measure cross-resistance. The tigecycline MICs of all resistant subpopulations and the levofloxacin MICs of 21 resistant subpopulations were each more than 4-fold higher than those of parental strains (**Table 3**). These findings indicated that resistant subpopulations had developed cross-resistance toward eravacycline, tigecycline, and levofloxacin.

**Table 3:** Change in antimicrobial activity to common antibiotics against resistant subpopulations.

Antibiotics	MIC of HP^a^ strains (mg/L) (n = 25)				MIC of RS strains (mg/L) (n = 25)				Fold change ≥4^b^ (n, %)
	Range	MIC_50_	MIC_90_	Num. of resistance(%)	Range	MIC_50_	MIC_90_	Num. of Resistance (%)	
Tigecycline	2–8	4	8	3 (12%)	16–128	32	64	25 (100%)	25 (100%)
Polymyxin B	1–2	1	2	0 (0%)	1–2	1	2	0 (0%)	0 (0%)
Meropenem	32–128	64	128	25 (100%)	32~>128	64	128	25 (100%)	0 (0%)
Levofloxacin	8–64	16	32	25 (100%)	64~>256	64	128	25 (100%)	21 (84%)
Gentamicin	128~>512	256	512	25 (100%)	128~>512	256	512	25 (100%)	0 (0%)
cefepime	64~>512	256	512	15 (100%)	128~>512	256	512	25 (100%)	0 (0%)

a HP, heteroresistant parental strains; RS, resistant subpopulations of eravacycline heteroresistant isolates.

b Number of resistant subpopulations with MIC values ≥4 times higher than heteroresistant parental strains.

### 3.4 Effects of the efflux pump inhibitor on eravacycline heteroresistance

To investigate the mechanisms of eravacycline heteroresistance, the effect of the EPIs on the eravacycline MIC of resistant subpopulations was determined first. The eravacycline MICs were significantly decreased by ≥4 fold with the addition of the PaβN or CCCP in 88% (22/25) or 64% (16/25) of the resistant subpopulations, respectively (**Table 4**). These findings indicated that EPIs could potentiate eravacycline activity in heteroresistant CRAB isolates. We further examined the incidence of eravacycline heteroresistance in CRAB isolates by performing a PAP test with the EPIs. Only four eravacycline heteroresistant strains were detected from 144 CRAB isolates after the addition of PaβN, and only nine heteroresistant strains were identified with the addition of CCCP, significantly fewer than those detected in the absence of EPIs (*P* < 0.05) (**Table 5**). These results suggested that EPIs can inhibit the formation of eravacycline heteroresistance in CRAB isolates.

**Table 4:** Eravacycline MICs against the resistant subpopulations in the absence or presence of efflux pump inhibitors.

Strain	MIC (mg/L)		Fold change^c^	MIC (mg/L)	Fold change^d^
	Eravacycline	Eravacycline +PaβN^b^		Eravacycline +CCCP	
CRAB-5-RS^a^	32	4	8	32	1
CRAB-9-RS	16	2	8	8	2
CRAB-11-RS	64	8	8	32	2
CRAB-14-RS	32	16	2	8	4
CRAB-21-RS	16	4	4	4	4
CRAB-24-RS	16	2	8	8	2
CRAB-38-RS	64	8	8	8	8
CRAB-44-RS	32	8	4	4	8
CRAB-49-RS	16	2	8	8	2
CRAB-50-RS	32	2	16	16	2
CRAB-53-RS	16	8	2	8	2
CRAB-58-RS	16	2	8	2	8
CRAB-64-RS	32	8	4	4	8
CRAB-68-RS	16	4	4	4	4
CRAB-77-RS	16	2	8	2	8
CRAB-82-RS	32	4	8	4	8
CRAB-84-RS	16	8	2	16	1
CRAB-87-RS	16	4	4	2	8
CRAB-90-RS	16	2	8	4	4
CRAB-92-RS	16	2	8	2	8
CRAB-102-RS	32	4	8	8	4
CRAB-108-RS	32	8	4	8	4
CRAB-113-RS	32	4	8	16	2
CRAB-127-RS	32	8	4	4	8
CRAB-139-RS	16	2	8	4	4

a RS, resistant subpopulations of eravacycline heteroresistant isolates.

b PaβN, Phe-Arg-β-naphthylamide (50 mg/L); CCCP, carbonyl cyanide m-chlorophenylhydrazone (16 mg/L).

c The fold change means the ratio of Eravacycline MIC and (Eravacycline +PaβN) MIC.

d The fold change means the ratio of Eravacycline MIC and (Eravacycline +CCCP) MIC.

**Table 5:** PAP test for eravacycline heteroresistance with PaβN or CCCP in CRAB isolates.

MIC of CRAB isolates (mg/L)	Positive results of PAP test^a^				
	Eravacycline	Eravacycline +PaβN^b^	*P*-value^c^	Eravacycline +CCCP	*P*-value
≤0.06 (4^d^)	0	0	/	0	/
0.12–0.25 (124)	18	2	<0.001	6	0.010
0.5–1 (12)	5	1	0.059	1	0.059
2–4 (4)	2	1	0.465	2	1.000
Total (144)	25	4	<0.001	9	0.003

a Eravacycline heteroresistance was performed in PAP assay with PAβN (50 mg/L) or CCCP (16 mg/L) added to the MH agar plates. Eravacycline heteroresistance is defined as an eravacycline -susceptible isolate (MIC ≤ 4 mg/L) with subpopulations growing on plates with ≥8 mg/L eravacycline, with a detection threshold of 20 CFU/mL.

b PaβN, Phe-Arg-β-naphthylamide; CCCP, carbonyl cyanide m-chlorophenylhydrazone.

c P value for the positive results of PAP test between Eravacycline + EPI and Eravacycline.

d Number of strains with different MIC values.

### 3.5 Eravacycline heteroresistance mechanism in CRAB isolates

**Figure 1:**
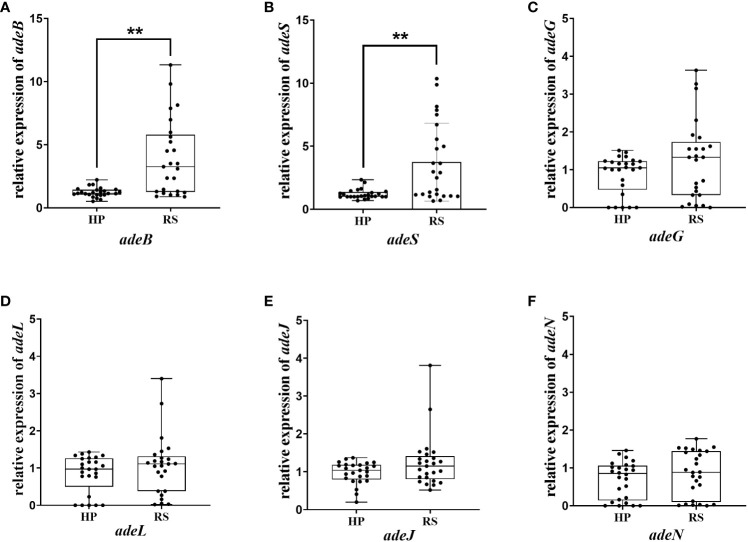
Relative transcription of the efflux pump gene in the heteroresistant parental strain and resistant subpopulations. Relative expression of *adeB* **(A)**, *adeS* **(B)**, *adeG* **(C)**, *adeL* **(D)**, *adeJ* **(E)** and *adeN* **(F)** were assessed by qRT-PCR analysis. The housekeeping gene, *rpoB*, was used as the endogenous reference gene. The heteroresistant parental strain CRAB-9-HP was used as the reference strain (expression = 1.0). All qRT-PCR experiments were carried out in triplicate. ∗∗* P <*0.05. HP, eravacycline heteroresistant parental strain; RS, resistant subpopulations of eravacycline heteroresistant strains.

The above results indicated that efflux pumps were involved in eravacycline heteroresistance. Thus, the relative expression of efflux pumps associated with eravacycline resistance was compared between eravacycline heteroresistant parental strains and their resistant subpopulations. The expression of *adeB* was significantly higher in 64% (16/25) of resistant subpopulations compared with the parental strains, and *adeS* expression was significantly increased in 56% (14/25) of resistant subpopulations (**Figure 1**). No significant differences were found in the expression of other efflux pump genes. These findings suggested that high expression of the efflux pump, AdeABC, and its regulator, AdeRS, may contribute to eravacycline heteroresistance. Genetic alterations of efflux pumps and ribosomes were then investigated in resistant subpopulations. An IS*Aba1* insertion was detected in *adeS* in 40% (10/25) of resistant subpopulations (**Table 6**), indicating that this insertion may be associated with eravacycline heteroresistance.

**Table 6:** Mutations of the efflux pump and ribosome genes in resistant subpopulations of eravacycline heteroresistant strains.

Resistant suspopulations	*adeR* ^a^	*adeS*	*adeL*	*adeN*	*rpsJ*
CRAB-5-RS^b^	W^c^	E121K	W	W	W
CRAB-9-RS	W	IS*Aba1*	W	W	W
CRAB-11-RS	W	F170S	W	W	W
CRAB-14-RS	W	IS*Aba1*	W	W	W
CRAB-21-RS	R13K	W	W	W	W
CRAB-24-RS	W	IS*Aba1*	W	W	W
CRAB-38-RS	W	W	W	W	W
CRAB-44-RS	W	W	W	W	W
CRAB-49-RS	W	IS*Aba1*	W	W	W
CRAB-50-RS	W	IS*Aba1*	W	W	W
CRAB-53-RS	W	W	W	W	W
CRAB-58-RS	N174K	W	W	W	W
CRAB-64-RS	W	W	W	W	W
CRAB-68-RS	W	W	W	W	W
CRAB-77-RS	W	IS*Aba1*	W	W	W
CRAB-82-RS	W	W	W	Q136N	W
CRAB-84-RS	W	W	W	W	W
CRAB-87-RS	W	IS*Aba1*	W	W	W
CRAB-90-RS	W	W	W	W	W
CRAB-92-RS	W	W	W	W	W
CRAB-102-RS	W	W	W	W	W
CRAB-108-RS	W	IS*Aba1*	W	W	W
CRAB-113-RS	W	W	R262Q	W	W
CRAB-127-RS	W	IS*Aba1*	W	W	W
CRAB-139-RS	N20D	IS*Aba1*	W	W	W

a Mutations were determined by PCR amplification and sequence alignment of the resistant subpopulations and parental strains.

b RS, resistant subpopulations of eravacycline heteroresistant isolates.

c W, wild-type of the gene.

### 3.6 Validation of the roles of AdeABC and AdeRS in eravacycline heteroresistance

**Figure 2:**
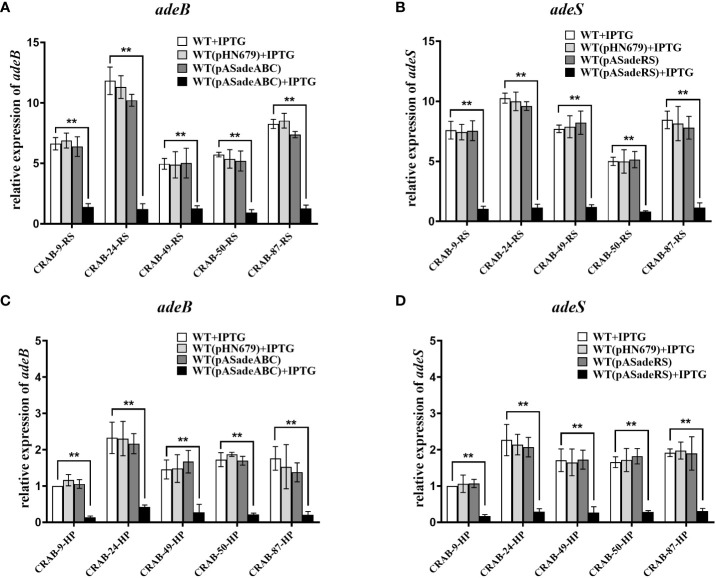
Transcriptional level of *adeB*/*adeS* in *adeABC-* and *adeRS*-silenced strains. Resistant subpopulations and parental strains transformed with pAS*adeABC* or pAS*adeRS* were grown in LB broth for 5 hours induced with or without 1 mM IPTG. Total RNA was extracted and the expressions of *adeB* **(A, C)** or *adeS* **(B, D)** were assessed by qRT-PCR. The housekeeping gene, *rpoB*, was used as an endogenous reference gene. CRAB-9-HP was used as the reference strain (expression = 1.0). All qRT-PCR experiments were carried out in triplicate. ** *P <*0.05. HP, eravacycline heteroresistant parental strain, RS, resistant subpopulations of eravacycline heteroresistant strains. WT, wild type of the strains. IPTG, isopropyl β-D-thiogalactoside.

To further confirm the role of the efflux pump, AdeABC, and its regulator, AdeRS, in the eravacycline heteroresistance of CRAB isolates, asRNA was used to individually silence the expression of *adeABC* and *adeRS* in eravacycline heteroresistant parental strains and their resistant subpopulations. The asRNA plasmids, pAS*adeABC* and pAS*adeRS*, were constructed and transformed into resistant subpopulations or parental strains (five strains per gene) that showed high expression of the target gene, respectively (**Supplementary Table 5**). The silencing efficacy of the candidate genes in the transformed strains was confirmed by qRT-PCR. After induction with 1.0 mM IPTG, the expression of *adeABC* and *adeRS* was downregulated by >80% more in the constructed strains than in the wildtype strains (**Figure 2**). The eravacycline MICs of resistant subpopulations decreased by 4- to 8-fold after silencing *adeABC* or *adeRS* (**Table 7**). Furthermore, PAP revealed that silencing *adeABC* or *adeRS* led to a loss of the eravacycline heteroresistant phenotype in 80% (4/5) or 60% (3/5) of parental strains, respectively (**Supplementary Table 9**). These results confirmed that high expression of AdeABC or AdeRS contributed to the formation of eravacycline heteroresistance in CRAB isolates.

**Table 7:** Effects on eravacycline MICs in resistant subpopulations after silencing *adeABC* or *adeRS*.

Transformed plasmid	Isolate	Eravacycline MIC (mg/L)^a^		
		Wildtype strain^b^	Vector control strain^c^	Derivative strain^d^
pAS*adeABC*	CRAB-9-RS	16	16	2
	CRAB-24-RS	16	8	2
	CRAB-49-RS	16	16	4
	CRAB-50-RS	32	16	4
	CRAB-87-RS	16	16	4
pAS*adeRS*	CRAB-9-RS	16	16	4
	CRAB-24-RS	16	16	4
	CRAB-49-RS	16	8	2
	CRAB-50-RS	32	16	4
	CRAB-87-RS	16	16	2

a Eravacycline MIC was detected in strains after induction with 1.0 mM IPTG.

b Wildtype strain, resistant subpopulation of eravacycline heteroresistant CRAB isolates.

c Vector control strain, resistant subpopulation transformed with the pHN679 vector.

d Derivative strain, resistant subpopulation transformed with the pASadeABC or pASadeRS plasmid.

## 4 Discussion

CRAB exhibits resistance to almost all commonly used antibiotics, which poses a significant threat to hospitalized patients. The available treatment options are extremely limited, with no option found to reduce mortality (which exceeds 40% in most studies) or substantially improve clinical outcomes (O'Donnell et al., 2021). Eravacycline confers excellent *in vitro* antimicrobial activity against CRAB, indicating its potential for use in the treatment of CRAB infections. While eravacycline heteroresistance in CRAB isolates is a serious issue in China, the mechanism of resistance has been poorly understood. This study investigated the characteristics and mechanisms of eravacycline heteroresistance in CRAB isolates to inform appropriate treatment options for CRAB infections.

In this study, eravacycline (MIC_90_ of 1 mg/L) showed better *in vitro* antimicrobial activity than tigecycline and polymyxins B, which is consistent with previous reports (Liu et al., 2022). However, 17.36% of the CRAB strains exhibited heteroresistance to eravacycline. Prior research has shown that more than half of the clinical *A. baumannii* isolates were heteroresistant to tigecycline (Jo and Ko, 2021). This suggests that heteroresistance may be prevalent in *A. baumannii* isolates treated with tetracycline antibiotics. However, the frequency of eravacycline heteroresistance in this study was significantly lower than tigecycline heteroresistance in Jo’s study, which indicates that tigecycline is more likely to develop heteroresistance in *A. baumannii* isolates than eravacycline. Risk of eravacycline heteroresistance has been shown in *Enterococcus faecalis*, *Staphylococcus aureus*, *Streptococcus agalactiae*, and *Klebsiella pneumoniae* (Zhang et al., 2018; Zheng et al., 2018b; Li et al., 2020; Wen et al., 2020). However, the current study is the first to report eravacycline heteroresistance in *A. baumannii*, with CRAB isolates exhibiting this trait while CSAB isolates did not. These results indicated that CRAB isolates are more likely to develop eravacycline heteroresistance. As the resistant subpopulations of heteroresistance occurs at a low frequency, they are often undetected during routine diagnostic processes and usually misclassified as susceptible strains (Stojowska-Swędrzyńska et al., 2021). Therefore, heteroresistance may lead to antibiotic treatment failure, persistent infection and even lethal outcomes (Juhász et al., 2017). Monitoring the frequency of heteroresistance can provide information about the potential risk of eravacycline resistance among CRAB isolates. In addition, the incidence of eravacycline heteroresistance in CRAB isolates increased with the patent of MIC value, which indicated that the frequency of eravacycline heteroresistance in *A. baumannii* may be MIC dependent (Kim et al., 2020; Jo and Ko, 2021). Thus, it is important to monitor strains that are close to the eravacycline resistance breakpoint (MIC of 4 mg/L) and make appropriate treatment decisions to avoid the emergence of heteroresistance in clinical practice. The eravacycline MICs of resistant subpopulations were restored to the susceptible level after serial passaging on antibiotic-free medium. This finding indicated that resistant subpopulations are reversible rather than a result of stable mutations, which is consistent with previous reports (Band et al., 2016).

Eravacycline heteroresistant strains mainly originate from the ICU and the burn and neurosurgery departments, where the patients are often immunocompromised. The emergence of heteroresistance and potential antibiotic treatment failure can put these patient populations at a serious health risk. Thus, preventive action is needed to prevent eravacycline heteroresistance in departments with a high incidence of infection. All eravacycline heteroresistant strains contain OXA-23, the most common carbapenemase in *A. baumannii* (Han et al., 2017), which is disseminated through transposons or plasmids (Zong et al., 2020). In current study, 72% of the eravacycline heteroresistant strains were ST208, one of the predominant clones in China (Jiang et al., 2021). Seruga et al. found that ST195 was the prevalent MLST of *A. baumannii* heteroresistant to colistin (Seruga Music et al., 2017). It indicates that the prevalent MLST of heteroresistant *A. baumannii* shows differences to various antibiotics. Previous reports have demonstrated that ST208 *A. baumannii* strains, which produce the OXA-23 enzyme, can spread rapidly and cause outbreaks in ICUs (Wang et al., 2021b). Our findings illustrated that *A. baumannii* ST208 producing OXA-23 carbapenemase exhibited heteroresistance to eravacycline, highlighting the need for strict ICU infection control interventions.

EPIs bind to efflux proteins, reducing the ability of the pumps to interact with their substrates and inhibiting multi-drug efflux (Jamshidi et al., 2017). Our data show that the eravacycline MICs were significantly decreased with the addition of the PaβN or CCCP in 88% or 64% of the resistant subpopulations, respectively. It indicates that the effect of PaβN to decrease eravacycline MIC in resistant subpopulations is better than CCCP, which may associate with the differences of their functional mechanism (Li et al., 2015). The current study showed that EPI addition could lower eravacycline MICs in resistant subpopulations and reduce eravacycline heteroresistance in parental strains. These findings indicate that efflux pumps may be involved in inducing eravacycline heteroresistance under antibiotic pressure. The resistance of *A. baumannii* to eravacycline and tigecycline is mainly associated with the overexpression of the RND-type efflux pumps, AdeABC, AdeFGH, and AdeIJK (Yang et al., 2019; Shi et al., 2020; Jo and Ko, 2021), which are controlled by the transcriptional regulators, AdeRS, AdeL, and AdeN (Gerson et al., 2018; Hua et al., 2021). Thus, qRT-PCR was used to assess the expression of these efflux pumps and their regulatory genes in the eravacycline heteroresistant parental strain and the resistant subpopulations. The expression of *adeABC* and *adeRS* were significantly upregulated in the resistant subpopulations, suggesting that eravacycline heteroresistance may be related to the high expression of AdeABC and AdeRS. These findings are similar to those reported in eravacycline resistance studies. Shi et al. found that a deletion mutation in the *adeS* gene could regulate high expression of the efflux pump, AdeABC, leading to eravacycline resistance in *A. baumannii* isolates (Shi et al., 2020).

Mutations in upstream regulatory genes of efflux pumps, including *adeRS*, *adeL*, and *adeN* and the ribosomal S10 protein encoding gene *rpsJ* are thought to contribute to tigecycline and eravacycline resistance in *A. baumannii* (Shi et al., 2020). To assess this, the current study performed genetic mutation analysis on these genes. The IS*Aba1* insertion was detected in *adeS* in 40% (10/25) of resistant subpopulations. This insertion sequence is a common genetic element present in the chromosomes and plasmids of *A. baumannii* that has been associated with resistance to various antibiotics (Sun et al., 2012). AdeS is a membrane-integrated sensor protein with histidine kinase domains that can transmit environmental stimuli to the response regulator, AdeR, to regulate the expression of the *adeABC* genes (Coyne et al., 2011). Sun et al. found that the *adeS* gene with an IS*Aba1* insertion can enhance *adeR* expression and upregulate *adeABC* expression in *A. baumannii* isolates (Sun et al., 2012). Thus, the current study assessed whether the IS*Aba1* insertion in *adeS* impacted the transcription of *adeABC.* The expression of *adeB* was significantly higher in all resistant subpopulations with the IS*Aba1* insertion in *adeS* than in the parental strains (**Supplementary Figure 1**), which are consistent with the findings of Sun et al (Sun et al., 2012). Besides, Jo et al. found that the upregulation of *adeABC* due to IS*Aba1* insertion in *adeS* might be the main heteroresistance mechanism of tigecycline in *A. baumannii* isolates (Jo and Ko, 2021). Our data and Jo’s finding suggest that the high expression of AdeABC efflux pumps may contribute to both eravacycline and tigecycline heteroresistance in CRAB isolates. Appropriate therapeutic options for CRAB infections should be choose as the two drugs share similar heteroresistant mechanism.

To further confirm the roles of the efflux pump, AdeABC, and its regulator, AdeRS, in eravacycline heteroresistant strains, asRNA was used to silence these genes in resistant subpopulations and their parental strains, respectively. Silencing *adeABC* or *adeRS* was able to restore sensitivity to eravacycline in resistant subpopulations and reduce eravacycline heteroresistance in parental strains. These findings indicated that high expression of AdeABC and AdeRS may contribute to eravacycline heteroresistance in CRAB isolates. The MIC values of eravacycline, tigecycline, and levofloxacin all increased in the resistant subpopulations, indicating potential cross-resistance between eravacycline and these two drugs. Prior reports have shown that high expression of AdeABC leads to tigecycline resistance (Ruzin et al., 2007), explaining the cross-resistance between eravacycline and tigecycline observed in this study. Mehdi et al. showed that the efflux pumps, EfrAB, EfmA, and EmeA, could induce fluoroquinolone resistance in *E. faecium* clinical isolates (Mirzaii et al., 2023) and our data suggest that high expression of AdeABC may affect fluoroquinolone resistance. Additional study is required to assess this further.

In conclusion, this study identified the emergence of eravacycline heteroresistance in CRAB isolates in China, with ST208 being the predominant clone. Eravacycline was shown to have cross-resistance with tigecycline and levofloxacin in resistance subpopulations. In addition, the high expression of AdeABC and AdeRS was found to contribute to eravacycline heteroresistance in CRAB isolates.

## Data availability statement

The original contributions presented in the study are included in the article/**Supplementary Material**. Further inquiries can be directed to the corresponding authors.

## Ethics statement

All procedures performed were approved by the Ethical Committee of Yangjiang People's Hospital and were in accordance with the 1964 Helsinki Declaration and its later amendments.

## Author contributions

Y-TL: Investigation, Methodology, Writing – original draft, Writing – review & editing. X-DC: Methodology, Validation, Writing – review & editing. Y-YG: Writing – review & editing, Methodology, Formal analysis. S-WL: Methodology, Writing – review & editing. M-ZW: Investigation, Writing – review & editing. J-BX: Investigation, Writing – review & editing. X-HW: Methodology, Writing – review & editing. G-HH: Resources, Writing – review & editing. X-XT: Software, Writing – review & editing. CZ: Supervision, Writing – review & editing. Z-WL: Supervision, Writing – review & editing, Conceptualization, Funding acquisition.
