# Supplementary material for: Emergence of eravacycline heteroresistance in carbapenem-resistant Acinetobacter baumannii isolates in China
Source: Front Cell Infect Microbiol. 2024 Mar 27;14:1356353. doi: 10.3389/fcimb.2024.1356353 (PMC11004246; doi:10.3389/fcimb.2024.1356353)
Supplement: Supplementary file 1 [file DataSheet_1.docx]

Supplementary Material

Emergence of Eravacycline Heteroresistance in Carbapenem-Resistant *Acinetobacter baumannii* Isolates in China

**Yi-tan Li^1+^, Xian-di Chen^1+^, Ying-yi Guo^2+^, Shan-wen Lin^1^, Ming-zhen Wang^1^, Jian-bo Xu^1^, Xiao-hu Wang^1^, Guo-hua He^1^, Xi-xi Tan^1^, Chao Zhuo^2*^, Zhi-wei Lin^1*^**

*** Correspondence:** Zhi-wei Lin: 422156321@qq.com and Chao Zhuo: chao_sheep@263.net

# Supplementary Data

# Supplementary Figures and Tables

## Supplementary Figures


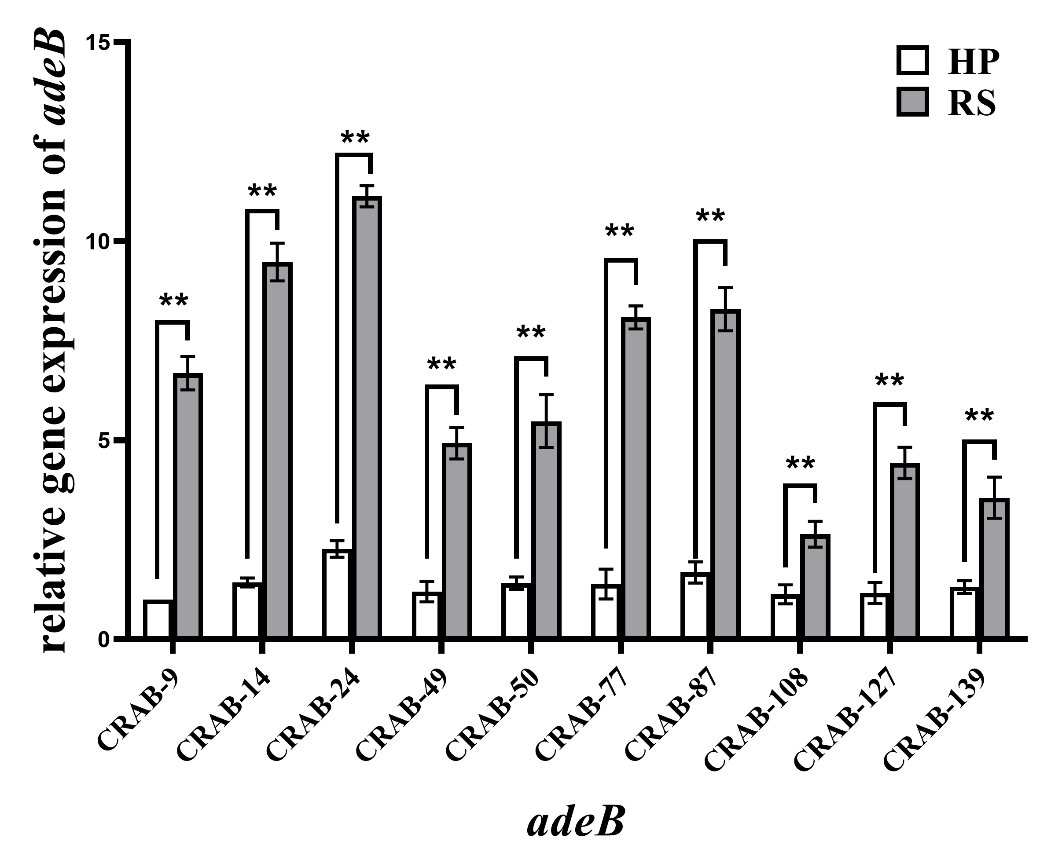


**Supplementary Figure 1.** Relative transcriptional analysis of *adeABC* in resistant subpopulations with IS*Aba1* insertions in the *adeS* compare to heteroresistant parental strain. Relative expression of *adeB* was assessed using qRT-PCR analysis. The housekeeping gene, *rpoB*, was used as the endogenous reference gene. CRAB-9-HP was used as the reference strain (expression = 1.0). All qRT-PCR experiments were carried out in triplicate. ** *P* <0.05. HP, eravacycline heteroresistant parental strain; RS, resistant subpopulations of eravacycline heteroresistant strains.

## Supplementary Tables

**Supplementary Table 1.** Primers used for the Oxford scheme of MLST determination in *A. baumannii*.

| **Target gene** | **Primer** | **Primer sequence (5'to3')** | **Amplicon size (bp)** | **Source** |
| --- | --- | --- | --- | --- |
| *gltA^a^* | *gltA-F* | AATTTACAGTGGCACATTAGGTCCC | 722 | (Bartual et al., 2005) |
|  | *gltA-R* | GCAGAGATACCAGCAGAGATACACG |  |  |
| *gyrB* | *gyrB-F* | TGAAGGCGGCTTATCTGAGT | 594 | (Bartual et al., 2005) |
|  | *gyrB-R* | GCTGGGTCTTTTTCCTGACA |  |  |
| *gdhB* | *gdhB-SF* | ACCACATGCTTTGTTATG | 774 | (Bartual et al., 2005) |
|  | *gdhB-SR* | GTTGGCGTATGTTGTGC |  |  |
| *recA* | *recA-F* | CCTGAATCTTCYGGTAAAAC | 425 | (Bartual et al., 2005) |
|  | *recA-R* | GTTTCTGGGCTGCCAAACATTAC |  |  |
| *cpn60* | *cpn60-F* | GGTGCTCAACTTGTTCGTGA | 640 | (Bartual et al., 2005) |
|  | *cpn60-R* | CACCGAAACCAGGAGCTTTA |  |  |
| *gpi* | *gpi-F* | GAAATTTCCGGAGCTCACAA | 456 | (Bartual et al., 2005) |
|  | *gpi-R* | TCAGGAGCAATACCCCACTC |  |  |
| *rpoD* | *rpoD-F* | ACCCGTGAAGGTGAAATCAG | 672 | (Bartual et al., 2005) |
|  | *rpoD-R* | TTCAGCTGGAGCTTTAGCAAT |  |  |

^a^*gltA*, citrate synthase; *gyrB*, DNA gyrase subunit B; *gdhB*, glucose dehydrogenase B; *recA*, homologous recombination factor; *cpn60*, 60-kDa chaperonin; *gpi*, glucose-6-phosphate isomerase; *rpoD*, RNA polymerase sigma factor.

**Supplementary Table 2.** Primers used for amplification of carbapenemase genes in eravacycline heteroresistant strains.

| **Target gene** | **Primer** | **Primer sequence (5'-3')** | **Amplicon size (bp)** | **Source** |
| --- | --- | --- | --- | --- |
| Carbapenemase resistance genes | | | | |
| *bla*_KPC_ | KPC-F | CGTCTAGTTCTGCTGTCTTG | 798 | (Hassan et al., 2021) |
|  | KPC-R | CTTGTCATCCTTGTTAGGCG |  |  |
| *bla*_IMP_ | IMP-F | CTACCGCAGCAGAGTCTTTG | 587 | (Hassan et al., 2021) |
|  | IMP-R | AACCAGTTTTGCCTTACCAT |  |  |
| *bla*_VIM_ | VIM-F | AGTGGTGAGTATCCGACAG | 261 | (Hassan et al., 2021) |
|  | VIM-R | ATGAAAGTGCGTGGAGAC |  |  |
| *bla*_SIM_ | SIM-F | TACAAGGGATTCGGCATCG | 570 | (Hassan et al., 2021) |
|  | SIM-R | TAATGGCCTGTTCCCATGTG |  |  |
| *bla*_NDM-1_ | NDM1-F | TGCCGAGCGACTTGGCCTTG | 379 | (Hassan et al., 2021) |
|  | NDM1-R | ACCGATGACCAGACCGCCCA |  |  |
| *bla*_OXA-23_ | OXA23-F | GATCGGATTGGAGAACCAGA | 501 | (Hassan et al., 2021) |
|  | OXA23-R | ATTTCTGACCGCATTTCCAT |  |  |
| *bla*_OXA-24_ | OXA24-F | GGTTAGTTGGCCCCCTTAAA | 246 | (Hassan et al., 2021) |
|  | OXA24-R | AGTTGAGCGAAAAGGGGATT |  |  |
| *bla*_OXA-48_ | OXA48-F | GCGTGGTTAAGGATGAACAC | 438 | (Hassan et al., 2021) |
|  | OXA48-R | CATCAAGTTCAACCCAACCG |  |  |
| *bla*_OXA-58_ | OXA58-F | AAGTATTGGGGCTTGTGCTG | 599 | (Hassan et al., 2021) |
|  | OXA58-R | CCCCTCTGCGCTCTACATAC |  |  |

**Supplementary Table 3.** Primers used for amplification of efflux pumps regulator and ribosomal gene.

| **Target gene** | **Primer** | **Primer sequence (5'-3')** | **Amplicon size (bp)** | **Source** |
| --- | --- | --- | --- | --- |
| *adeS* | *adeS-F* | GCGCTGCGGTTACAGCTTATAT | 1387 | This study |
|  | *adeS-R* | GAACCGTAGATAGCCATGTGAGT |  |  |
| *adeR* | *adeR-F* | GAGCTTAAGCTAATCCAGCC | 991 | This study |
|  | *adeR-R* | AAGTGTGGAGTAAGTGTGGAG |  |  |
| *adeL* | *adeL-F* | AAGATGGCAGACAGTGCAAACT | 1325 | This study |
|  | *adeL-R* | AAGCAATGATCGGTATTGACG |  |  |
| *adeN* | *adeN-F* | ACAATTATCTGTTGTTGGCTGG | 807 | This study |
|  | *adeN-R* | ACATACCAATGACCATCGTT |  |  |
| *rpsJ* | *rpsJ-F* | ACAGTCGTGGCATCGATCACG | 668 | This study |
|  | *rpsJ-R* | AGTTACGCGAGACTCGCGACGT |  |  |

**Supplementary Table 4.** Primers for qRT-PCR in this study.

| **Target gene** | **Primer^a^** | **Primer sequence (5'-3')** | **Amplicon size (bp)** | |
| --- | --- | --- | --- | --- |
| *rpoB* | *qrpoB-F* | CAGAAGAGAAGAACAAGTTAT | 103 |  |
|  | *qrpoB -R* | CGTGAAGAAGCATTAGTAG |  |  |
| *adeB* | *qadeB-F* | GGAATAAGGCACCACAACAAT | 75 |  |
|  | *qadeB-R* | CGAAGTTAGGAATACCAGCAATAC |  |  |
| *adeS* | *qadeS-F* | TTGCTGGTTCTCTACTAAG | 156 |  |
|  | *qadeS -R* | ACGCCTATAACGATATTACA |  |  |
| *adeG* | *qadeG-F* | GTTAATATCTGCTGATGTGTAA | 91 |  |
|  | *qadeG -R* | GGTGCCATTATCTTCATTG |  |  |
| *adeL* | *qadeL-F* | AATATGGCGAACCTACCT | 169 |  |
|  | *qadeL-R* | ATATAAGCATCACCGTCATT |  |  |
| *adeJ* | *qadeJ-F* | AACTACAACAGCATAGAG | 143 |  |
|  | *qadeJ-R* | AAGAACCAAGCAATAATATC |  |  |
| *adeN* | *qadeN-F* | CCATAATCATTCGCCATTCAA | 114 |  |
|  | *qadeN -R* | CTCAAGCCTTACTCATATCTCA |  |  |

^a^ Primers for qRT-PCR were designed by Beacon designer software 8.14.

**Supplementary Table 5.** Bacterial strains and plasmids used in the antisense RNA silencing experiment.

| **Name** | **Description^a^** | **Source** |
| --- | --- | --- |
| **Bacterial Strains** |  |  |
| *A. baumannii* |  |  |
| CRAB-9-RS | Resistance suspopulation of eravacycline heteroresistant isolate CRAB-9 | this study |
| CRAB-24-RS | Resistance suspopulation of eravacycline heteroresistant isolate CRAB-24 | this study |
| CRAB-49-RS | Resistance suspopulation of eravacycline heteroresistant isolate CRAB-49 | this study |
| CRAB-50-RS | Resistance suspopulation of eravacycline heteroresistant isolate CRAB-50 | this study |
| CRAB-87-RS | Resistance suspopulation of eravacycline heteroresistant isolate CRAB-87 | this study |
| CRAB-9-HP | eravacycline heteroresistant parental isolate CRAB-9 | People’s Hospital of Yangjiang |
| CRAB-24-HP | eravacycline heteroresistant parental isolate CRAB-24 | People’s Hospital of Yangjiang |
| CRAB-49-HP | eravacycline heteroresistant parental isolate CRAB-49 | People’s Hospital of Yangjiang |
| CRAB-50-HP | eravacycline heteroresistant parental isolate CRAB-50 | People’s Hospital of Yangjiang |
| CRAB-87-HP | eravacycline heteroresistant parental isolate CRAB-87 | People’s Hospital of Yangjiang |
| CRAB-9-RS (pAS*adeABC*) | CRAB-9-RS introduced with plasmid pAS*adeABC* | this study |
| CRAB-24-RS (pAS*adeABC*) | CRAB-24-RS introduced with plasmid pAS*adeABC* | this study |
| CRAB-49-RS (pAS*adeABC*) | CRAB-49-RS introduced with plasmid pAS*adeABC* | this study |
| CRAB-50-RS (pAS*adeABC*) | CRAB-50-RS introduced with plasmid pAS*adeABC* | this study |
| CRAB-87-RS (pAS*adeABC*) | CRAB-87-RS introduced with plasmid pAS*adeABC* | this study |
| CRAB-9-RS (pAS*adeRS*) | CRAB-9-RS introduced with plasmid pAS*adeRS* | this study |
| CRAB-24-RS (pAS*adeRS*) | CRAB-24-RS introduced with plasmid pAS*adeRS* | this study |
| CRAB-49-RS (pAS*adeRS*) | CRAB-49-RS introduced with plasmid pAS*adeRS* | this study |
| CRAB-50-RS (pAS*adeRS*) | CRAB-50-RS introduced with plasmid pAS*adeRS* | this study |
| CRAB-87-RS (pAS*adeRS*) | CRAB-87-RS introduced with plasmid pAS*adeRS* | this study |
| CRAB-9-HP (pAS*adeABC*) | CRAB-9-HP introduced with plasmid pAS*adeABC* | this study |
| CRAB-24-HP (pAS*adeABC*) | CRAB-24-HP introduced with plasmid pAS*adeABC* | this study |
| CRAB-49-HP (pAS*adeABC*) | CRAB-49-HP introduced with plasmid pAS*adeABC* | this study |
| CRAB-50-HP (pAS*adeABC*) | CRAB-50-HP introduced with plasmid pAS*adeABC* | this study |
| CRAB-87-HP (pAS*adeABC*) | CRAB-87-HP introduced with plasmid pAS*adeABC* | this study |
| CRAB-9-HP (pAS*adeRS*) | CRAB-9-HP introduced with plasmid pAS*adeRS* | this study |
| CRAB-24-HP (pAS*adeRS*) | CRAB-24-HP introduced with plasmid pAS*adeRS* | this study |
| CRAB-49-HP (pAS*adeRS*) | CRAB-49-HP introduced with plasmid pAS*adeRS* | this study |
| CRAB-50-HP (pAS*adeRS*) | CRAB-50-HP introduced with plasmid pAS*adeRS* | this study |
| CRAB-87-HP (pAS*adeRS*) | CRAB-87-HP introduced with plasmid pAS*adeRS* | this study |
| *E. coli* |  |  |
| DH5α | *supE44 △lacU169 hsdR17 recA1 endA1 gyrA96 thi-1 relA1* | Invitrogen |
| **Plasmids** |  |  |
| pHN679 | *P_trc_* - MCS-PT7 - *T_rrnB_*, *lacI^q^*, *Chl^r^*, *Kan^r^*, pACYC184 *ori* (p15A *ori*)^a^ | (Zheng et al., 2018) |
| pAS*adeABC* | pHN679 inserted with the asRNA silencing sequence of *adeABC* | this study |
| pAS*adeRS* | pHN679 inserted with the asRNA silencing sequence of *adeRS* | this study |

^a^*Chl^r^*, chloramphenicol resistance, *Kan^r^*, kanamycin resistance.

**Supplementary Table 6.** Primers used for construction of antisense RNA silencing strains.

| **Primers** | **Sequences（5’-3’）** | **Product**  **Length (bp)** | **Underline**  **(enzyme site)** |
| --- | --- | --- | --- |
| Construction of the *adeABC* and *adeRS* silencing plasmids | | | |
| ASadeABC-F | CCCAAGCTTTCACTAGGTTTGGACAGTATG | 168 | HindIII |
| ASadeABC-R | CGCGGATCCCGACTGCGGTTGAATGCTTAA |  | BamHI |
| ASadeRS-F | CCCAAGCTTGAAGCACTTTCTATAGCCAG | 166 | HindIII |
| ASadeRS-R | CGCGGATCCGAATAACACTCATGCCTTCA |  | BamHI |
| Verification of the silencing plasmids | | | |
| IDASadeABC-F | TCTATTGGGCTGATATTACAG | 785 |  |
| IDASadeABC-R | GTTGCGCGAGAAGATTGTGCA |  |  |
| IDASadeRS-F | TTGTGGTAGAAGATGACTACG | 793 |  |
| IDASadeRS-R | CAGCGGATAGTTAATGATCAG |  |  |

**Supplementary Table 7.** The susceptibilities of eravacycline and other antibiotics for 147 CRAB and 140 CSAB clinical strains.

| **Antibiotic** | **MIC (mg/L)** | | |  | **No.(%) of isolates and their susceptibility category** | | |
| --- | --- | --- | --- | --- | --- | --- | --- |
|  | **Range** | **MIC_50_** | **MIC_90_** |  | **S** | **I** | **R** |
| CRAB^a^(n=147) |  |  |  |  |  |  |  |
| Eravacycline^b^ | 0.06~16 | 0.25 | 1 |  | 142 (96.6) | 2 (1.4) | 3 (2.0) |
| Tigecycline | 0.12~64 | 1 | 2 |  | 114 (77.6) | 26 (17.7) | 7 (4.8) |
| Polymyxin B | 0.25~＞64 | 1 | 2 |  | 144 (98.0) | / | 3 (2.0) |
| Minocycline | 1~128 | 4 | 8 |  | 91 (61.9) | 35 (23.8) | 21 (14.3) |
| Tetracycline | 32~＞128 | ＞128 | ＞128 |  | 0 (0.0) | 0 (0.0) | 147 (100.0) |
| Meropenem | 8~＞128 | ＞128 | ＞128 |  | 0 (0.0) | 0 (0.0) | 147 (100.0) |
| Levofloxacin | 4~＞128 | ＞128 | ＞128 |  | 2 (1.4) | 5 (3.4) | 140 (95.2) |
| Gentamicin | 0.5~＞128 | 128 | ＞128 |  | 3 (2.0) | 1 (0.7) | 143 (97.3) |
| Ceftriaxone | 32~＞128 | ＞128 | ＞128 |  | 0 (0.0) | 2 (1.4) | 145 (98.6) |
| Ceftazidime | 16~＞128 | ＞128 | ＞128 |  | 0 (0.0) | 1 (0.7) | 146 (99.3) |
| Cefepime | 8~＞128 | ＞128 | ＞128 |  | 2 (1.4) | 4 (2.7) | 141 (95.9) |
| Piperacillin/Tazobactam | 16/4~＞128/4 | ＞128/4 | ＞128/4 |  | 1 (0.7) | 1 (0.7) | 145 (98.6) |
| Ampicillin/Sulbactam | 16/8~＞128/64 | ＞128/64 | ＞128/64 |  | 0 (0.0) | 5 (3.4) | 142 (96.6) |
| CSAB(n=140) |  |  |  |  |  |  |  |
| Eravacycline | 0.01~8 | 0.12 | 0.5 |  | 137 (97.9) | 2 (1.4) | 1 (0.7) |
| Tigecycline | 0.12~32 | 0.25 | 0.5 |  | 129 (92.1) | 6 (4.3) | 5 (3.6) |
| Polymyxin B | 0.25~4 | 1 | 2 |  | 138 (98.6) | / | 2 (1.4) |
| Minocycline | 0.12~32 | 2 | 4 |  | 119 (85.0) | 12 (8.6) | 9 (6.4) |
| Tetracycline | 0.5~＞128 | 2 | 4 |  | 97 (69.3) | 7 (5.0) | 36 (25.7) |
| Meropenem | 0.125~2 | 0.5 | 1 |  | 140 (100.0) | 0 (0.0) | 0 (0.0) |
| Levofloxacin | 0.06~16 | 0.25 | 0.5 |  | 123 (87.9) | 7 (5.0) | 10 (7.1) |
| Gentamicin | 0.25~64 | 1 | 2 |  | 124 (88.6) | 10 (7.1) | 6 (4.3) |
| Ceftriaxone | 0.25~32 | 4 | 8 |  | 128 (91.4) | 12 (8.6) | 0 (0.0) |
| Ceftazidime | 0.5~＞64 | 4 | 8 |  | 135 (96.4) | 2 (1.4) | 3 (2.1) |
| Cefepime | 0.5~＞64 | 2 | 4 |  | 136 (97.1) | 3 (2.1) | 1 (0.7) |
| Piperacillin/Tazobactam | 1/4~＞128/4 | 2/4 | 4/4 |  | 127 (90.7) | 11 (7.9) | 2 (1.4) |
| Ampicillin/Sulbactam | 1/0.5~＞64/32 | 2/1 | 4/2 |  | 126 (90.0) | 11 (7.9) | 3 (2.1) |

^a^CRAB, carbapenem-resistant *Acinetobacter baumannii*; CSAB, carbapenem-susceptible *A. baumannii.*

^b^As *Acinetobacter baumannii* MIC breakpoints for eravacycline and tigecycline have not yet been established by CLSI and FDA, this study categorized the MIC values into three levels based on reported breakpoints(Marchaim et al., 2014; Abdallah et al., 2015): ≤2 mg/L (sensitive, S), 4 mg/L (intermediate, I), and ≥8 mg/L (resistant, R).

**Supplementary Table 8.** Changes of eravacycline MICs in resistant subpopulations of heteroresistance isolates after serial passaging in antibiotic-free medium.

| **Strains** | **Eravacycline MIC of each passage(mg/L)** | | | | | | |
| --- | --- | --- | --- | --- | --- | --- | --- |
|  | **0** | **5** | **10** | **15** | **20** | **25** | **30** |
| CRAB-5-RS^a^ | 32 | 16 | 8 | 8 | 8 | 4 | 4 |
| CRAB-9-RS | 16 | 8 | 4 | 4 | 4 | 2 | 2 |
| CRAB-14-RS | 32 | 16 | 8 | 4 | 4 | 4 | 2 |
| CRAB-21-RS | 16 | 8 | 4 | 4 | 4 | 2 | 2 |
| CRAB-24-RS | 16 | 8 | 4 | 2 | 2 | 2 | 1 |
| CRAB-44-RS | 32 | 16 | 8 | 4 | 2 | 2 | 2 |
| CRAB-49-RS | 16 | 8 | 4 | 2 | 2 | 1 | 1 |
| CRAB-58-RS | 16 | 8 | 4 | 2 | 1 | 1 | 1 |
| CRAB-77-RS | 16 | 8 | 8 | 4 | 2 | 2 | 1 |
| CRAB-108-RS | 32 | 16 | 8 | 4 | 2 | 2 | 1 |

^a^RS, resistant subpopulation of eravacycline heteroresistant isolates.

**Supplementary Table 9.** The frequency of eravacycline heteroresistance in parental strains after silencing *adeABC* or *adeRS.*

| **Transformed**  **plasmids** | **Isolate** |  | **PAP test results^a^** | | |
| --- | --- | --- | --- | --- | --- |
|  |  |  | **Wildtype strain^b^** | **Vector control strain^c^** | **Derivative strain^d^** |
| pAS*adeABC* | CRAB-9 |  | + | + | – |
|  | CRAB-24 |  | + | + | – |
|  | CRAB-49 |  | + | + | – |
|  | CRAB-50 |  | + | + | – |
|  | CRAB-87 |  | + | + | + |
| pAS*adeRS* | CRAB-9 |  | + | + | + |
|  | CRAB-24 |  | + | + | – |
|  | CRAB-49 |  | + | + | – |
|  | CRAB-50 |  | + | + | + |
|  | CRAB-87 |  | + | + | – |

^a^Eravacycline heteroresistance is defined as an eravacycline-susceptible isolate (MIC ≤ 4 mg/L) with subpopulations growing in the presence ≥8 mg/L eravacycline, with a detection threshold of 20 CFU/mL. +, positive; –, negative.

^b^Wildtype strain, eravacycline heteroresistant parental strains.

^c^Vector control strain, parental strain transformed with the pHN679 vector.

^d^Derivative strain, parental strain transformed with the pAS*adeABC* or pAS*adeRS* plasmid.

# References

Abdallah, M., Olafisoye, O., Cortes, C., Urban, C., Landman, D., and Quale, J. (2015). Activity of eravacycline against Enterobacteriaceae and Acinetobacter baumannii, including multidrug-resistant isolates, from New York City. *Antimicrob Agents Chemother* 59(3)**,** 1802-1805. doi: 10.1128/aac.04809-14.

Bartual, S.G., Seifert, H., Hippler, C., Luzon, M.A., Wisplinghoff, H., and Rodríguez-Valera, F. (2005). Development of a multilocus sequence typing scheme for characterization of clinical isolates of Acinetobacter baumannii. *J Clin Microbiol* 43(9)**,** 4382-4390. doi: 10.1128/jcm.43.9.4382-4390.2005.

Hassan, R.M., Salem, S.T., Hassan, S.I.M., Hegab, A.S., and Elkholy, Y.S. (2021). Molecular characterization of carbapenem-resistant Acinetobacter baumannii clinical isolates from Egyptian patients. *PLoS One* 16(6)**,** e0251508. doi: 10.1371/journal.pone.0251508.

Marchaim, D., Pogue, J.M., Tzuman, O., Hayakawa, K., Lephart, P.R., Salimnia, H., et al. (2014). Major variation in MICs of tigecycline in Gram-negative bacilli as a function of testing method. *J Clin Microbiol* 52(5)**,** 1617-1621. doi: 10.1128/jcm.00001-14.

Zheng, J.X., Lin, Z.W., Chen, C., Chen, Z., Lin, F.J., Wu, Y., et al. (2018). Biofilm Formation in Klebsiella pneumoniae Bacteremia Strains Was Found to be Associated with CC23 and the Presence of wcaG. *Front Cell Infect Microbiol* 8**,** 21. doi: 10.3389/fcimb.2018.00021.
